# Retinoblastoma Presentation and Survival: A four-year analysis from a tertiary care hospital

**DOI:** 10.12669/pjms.36.ICON-Suppl.1720

**Published:** 2020-01

**Authors:** Nida Zia, Ahmer Hamid, Sundus Iftikhar, Muhammad Hamza Qadri, Anzal Jangda, Muhammad Rahil Khan

**Affiliations:** 1 Nida Zia, MCPS. Department of Peads Oncology, The Indus Hospital, Karachi, Pakistan; 2 Ahmer Hamid, FCPS. Department of Peads Oncology, The Indus Hospital, Karachi, Pakistan; 3 Sundus Iftikhar, MPhil (Statistics), Indus Hospital Research Center, The Indus Hospital, Karachi, Pakistan; 4 Muhammad Hamza Qadri, MBBS. Ziaudddin University, Karachi, Pakistan; 5 Anzal Jangda, MBBS. Ziaudddin University, Karachi, Pakistan; 6 Muhammad Rahil Khan, FCPS (Pediatric Hematology Oncology), Department of Peads Oncology, The Indus Hospital, Karachi, Pakistan

**Keywords:** Retinoblastoma, Outcome, Pediatric, Clinical features

## Abstract

**Objective:**

To study the clinical presentation, treatment, and outcome of Retinoblastoma (Rb) in a tertiary care hospital of Pakistan.

**Methods:**

A retrospective study was conducted in the Department of Pediatric Hematology Oncology, The Indus Hospital (TIH), Karachi from 1st June 2013 to 30th June 2017. Data including patients’ demography, clinical symptoms and duration, laterality, extent of the tumor, type of treatment, relapse, and final outcome were extracted and evaluated with respect to progression and survival.

**Results:**

A total of 93 patients were included; 34.4% were boys. The median age at presentation was 30 months. Leukocoria was the commonest symptom (61.3%), followed by proptosis (37.6%). Unilateral disease was seen in 59.1%, extraocular tumors in 43.5% and metastasis in 28.1%. Enucleation was performed on 46.2%, chemotherapy given to 80.6% and external beam radiation therapy to 29.3% patients.

**Conclusion:**

Delayed presentation, recurrent disease, extraocular disease and metastasis on presentation were factors affecting outcome in our cohort. Awareness about the early warning signs and symptoms in both public and health professionals for early recognition and timely management are mandatory to decrease morbidity and mortality.

## INTRODUCTION

Retinoblastoma (Rb) is the most common intraocular malignancy in children and accounts for 3% of pediatric (age <15 years) cancers.^1^ In the heritable genetic form, chromosome-13 mutation in the RB1 gene leads to Rb.^1^ The global prevalence of Rb is estimated to be one in 16,000 to 18,000 births per year with an incidence of up to 8000 cases annually.^2^ The World Health Organization (WHO) reports that 66% of children are diagnosed before their second year and 95% are diagnosed before age five.^2^ The incidence of retinoblastoma in Karachi, Pakistan is reported as 4 in 100,000 children under five years of age and 2.4 in 100,000 under age 10.^3^,^4^ The prevalence of unilateral and bilateral Rb is 64.07% and 35.93% respectively.^3^ In Asia-Pacific, of the 10 countries accounting for 90% incidence of Rb in the region, India is ranked highest whilst Pakistan is 6^th^.^5^

Rb is potentially curable with a very high disease-free survival. In the United States, the 5-year survival rate improved from 92.3% to 96.5% between 1975 and 2004.^2^,^6^ In Europe, the 5-year, 10-year and 18-year survival rate of Rb was 93%, 89%, and 86% respectively.^7^ However, the estimated survival rate ranges between 23% to 70% in lower income countries, and 60 to 92% in middle income countries.^8^ One of the main reasons behind this low survival rate is delayed presentation.^9^

The most common presenting feature of Rb is leukocoria (white pupillary reflex) which may be accompanied by strabismus (eye malalignment).^2^,^3^ As the condition progresses, children may present with buphthalmos (eyeball enlargement), orbital involvement or metastasis.^10^ Laterality and extent of the disease determines treatment i.e. use of focal intraocular therapy (intra-arterial/ intravitreal) or systemic chemotherapy, enucleation or radiotherapy.^11^

The purpose of this study was to analyze outcomes of Rb at The Indus Hospital, Karachi (TIH). Our centre is a not-for-profit, charity based hospital in Karachi serving a large number of low-income communities from the entire country. This will enable effective strategies and recommendations at national level to improve survival of these patients.

## METHODS

We conducted a four-year retrospective clinical chart review on all pediatric patients (up to age 15 years) with Rb, treated between 1st June 2013 to 30^th^ June 2017. The study was approved under IRB number: IRD_IRB_2018_01_002. The data were retrieved from the Electronic Medical Record system of TIH. Data included age, gender, clinical symptoms and their duration, laterality, extent of the disease (localized vs metastatic) and treatment modality (chemotherapy, radiotherapy, surgical enucleation, focal laser or cryotherapy). We collected the initial treatment status (treated or abandoned), relapse, and final outcome (alive – on/off treatment, dead).

Data were entered and analyzed using SPSS version 24.0. Mean ± SD or median (inter-quartile range, IQR) was computed as appropriate for age and duration of symptoms. Frequencies and percentages were computed for categorical variables. Univariate and multivariable logistic regression (LR) analyses were performed to assess the risk factors associated with mortality. Variables with p-value < 0.25 and of biological significance were included in the final multivariable analysis. Backward LR elimination method was applied to build the model. Statistical significance was accepted when p-value <0.05.

## RESULTS

**Fig.1:**
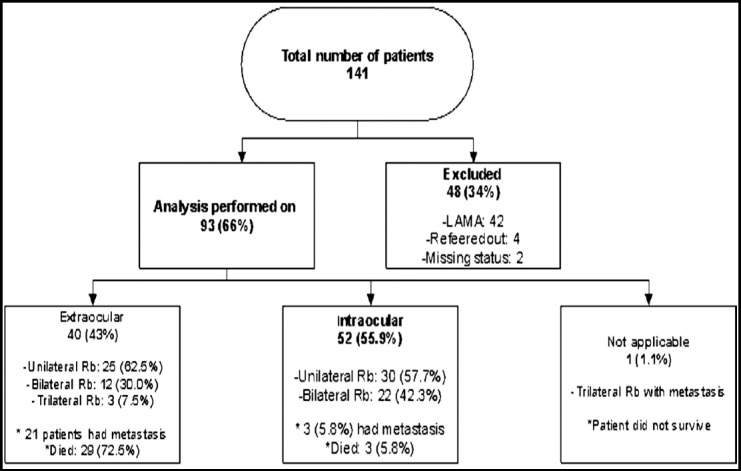
Flowchart depicting the distribution of disease.

During the study period, a total of 141 retinoblastoma patients were seen at TIH. The analysis was performed on 93 patients who completed treatment or died under treatment. Those patients who left against medical advice (n=48) or were transferred were excluded from the study (Fig.1).

From June 2013 to May 2014, a total of 30 cases of Rb were reported out; recurrent disease was found in 6 (20%). The following year, 23 cases were reported and 30.4% had recurrent disease. From June 2015 to May 2016, there were 11 cases with 23.9% recurrences. Between June 2016 to June 2017, 40 cases reported; 17.5% with disease recurrence. Overall 28% of cases had recurrence of disease over 4-years (Table-I).

**Table I:** Characteristics of the study patients.

Alive n=60	Dead n=33		P-value
Age (Month)^§^	30 (16.5 - 40.5)	42 (30 – 63)		0.001**^ɫ^
Treatment Delay(Month)^§^	2.5 (0.9 - 12)	7.5 (3.3 - 23)		0.003**^ɫ ^
*Gender; n (%)*				
Male	22 (36.7)	10 (30.3)		0.537^†^
Female	38 (63.3)	23 (69.7)	
*Leukokoria; n (%)*				
No	16 (26.7)	20 (60.6)		0.001**^†^
Yes	44 (73.3)	13 (39.4)	
*Proptosis; n (%)*				
No	47 (78.3)	11 (33.3)		0.000**^†^
Yes	13 (21.7)	22 (66.7)	
*Vision Loss; n (%)*				
No	48 (80)	24 (72.7)		0.422^†^
Yes	12 (20)	9 (27.3)	
*Squinting; n (%)*				
No	51 (85)	32 (97)		0.091^ⱡ^
Yes	9 (15)	1 (3)	
*Red Eye; n (%)*				
No	53 (88.3)	27 (81.8)		0.533^ⱡ^
Yes	7 (11.7)	6 (18.2)	
*Laterality; n (%)*				
BL	25 (41.7)	9 (27.3)		0.014*^†^
TL	0 (0)	4 (12.1)	
UL	35 (58.3)	20 (60.6)	
*Metastasis; n (%)*				
No	53 (94.6)	11 (33.3)		0.000**^†^
Yes	3 (5.4)	22 (66.7)	
*Extraocular; n (%)*				
No	49 (81.7)	3 (9.4)		0.000**^†^
Yes	11 (18.3)	29 (90.6)	
*Chemo; n (%)*				
No	14 (23.3)	4 (12.1)		0.190^†^
Yes	46 (76.7)	29 (87.9)	
*Enucleation (not including patients who already underwent enucleation somewhere else); n (%)*				
No	20 (33.3)	30 (90.9)		0.000**^†^
Yes	40 (66.7)	3 (9.1)	
*Recurrent Disease; n (%)*				
No	53 (88.3)	14 (42.4)		0.000**^†^
Yes	7 (11.7)	19 (57.6)	
*EBRT; n (%)*				
No	48 (81.4)	17 (51.5)		0.003*^†^
Yes	11 (18.6)	16 (48.5)	

* P-value<0.05,

** P-value<0.0001,

† Pearson Chi-Square test, †Fisher’s Exact test, ɫMann-Whitney U test.

In this cohort, up to two-thirds of patients (64.5%) survived. The median age at presentation was 30 months (IQR 18 – 42m). Boys constituted 34.4% (32/93). The median duration of symptoms prior to seeking medical care was 4 months (IQR 1 – 12 months). Presenting clinical features were leukocoria (61.3%), proptosis (37.6%), vision loss (22.6%), squint (10.8%) and red-eye (14%). Most (n=55, 59.1%) presented with unilateral Rb and 28.1% of patients had metastasis, 43.5% of which was extraocular disease. Chemotherapy was given to 80.6%, external beam radiation therapy (EBRT) to 29.3% and enucleation performed in 46.2% patients (Table-I).

Patients who died were older than survivors (42 vs 30 months, p=0.001). No significant association was observed between survival status and gender (p=0.537). Moreover, non-survivors were delayed in seeking treatment as compared to those who survived (7.5 vs 2.5 months, p=0.039). A higher proportion of bilateral disease was present in surviving patients versus non-survivors (41.7% vs 27.3%, p-value=0.014). Similarly, the proportion of metastatic and extraocular disease was higher in non-survivors in contrast to survivors (66.7% vs. 5.4%, p=0.000 and 90.6% vs. 18.3%, p=0.000 respectively). Furthermore, patients who had timely enucleation of their affected eye had a better survival rate (93% vs. 40%, p=0.000). Conversely, patients with recurrent disease on presentation after enucleation elsewhere had a higher relapse rate (73.1% vs 20.9%, p=0.000). A greater number of non-survivors required ERBT compared to patients who survived (48.5% vs. 18.6%) respectively (Table-I).

In univariate analysis, older patients and those with delayed presentation had 4% higher likelihood of death (95% CI=1.02-1.06 vs 1.002-1.08 respectively. Patients with extraocular disease, recurrent disease at presentation, metastasis, and proptosis had 43.1 (95% CI=11.1-167.2), 10.3 (95% CI=3.6-29.3), 35.3 (95% CI=8.9-139), and 7.2 (95% CI=2.8-18.7) times higher odds of death respectively. Patients presenting with leukocoria were found to have 80% less likelihood of progression to death (OR: 0.2, 95% CI 0.1 – 0.6. Table-II

In multivariable analysis, extraocular disease (OR: 11.9, 95% CI=2.1-66.1), recurrent disease at presentation (OR: 5.2, 95% CI=1.0-26.9), and metastasis (OR: 20.6, 95% CI=3.6-17.8) were found to be significantly associated with death adjusting for chemotherapy and gender (Table-II).

**Table II:** Risk factors associated with death.

	Univariate binary logistic regression		Multivariable binary logistic regression	

	Dead		Dead	

Variables	Crude OR	95% CI	adjusted OR	95% CI
Age in month	1.04	1.02 - 1.06	-	-
Delay in seeking treatment (months)	1.04	1.002 - 1.08	-	-
*Gender*				
Male	1.3	0.5 - 3.3	1.9	0.4 - 9.2
Female	Ref	ref		
*Laterality*				
UL	1.6	0.6 - 4.1	-	-
BL	Ref	-	-	
*Treatment modalities*				
*Chemotherapy*				
Yes	2.2	0.7 - 7.4	5.1	0.348 - 73.8
No	Ref	ref		
*Treatment group*				
*Extracoular*				
Yes	43.1	11.1 - 167.2	11.9	2.1 - 66.1
No	Ref	ref		
*Recurrent Disease*				
Yes	10.3	3.6 - 29.3	5.2	1.0 - 26.9
No	Ref	ref		
*Metastasis*				
Yes	35.3	8.9 - 139	20.6	3.6 - 17.8
No	Ref	ref		
*Leucokoria*				
Yes	0.2	0.1 - 0.6	-	-
No	Ref	-	-	
*Proptosis*				
Yes	7.2	2.8 - 18.7	-	-
No	Ref	-	-	
*Vision Loss*				
Yes	1.5	0.6 - 4.1	-	-
No	Ref	-	-	
*Red eye*				
Yes	1.7	0.5 - 5.5	-	-
No	Ref	-	-	
*Squinting*				
Yes	0.2	0.02 - 1.5	-	-
No	Ref	-	-	

Reference category: Alive;

*P-value<0.05,

**P-value<0.0001.

## DISCUSSION

The Pediatric Oncology Department, TIH, registers 800 to 900 new cases of cancer per year, of which retinoblastoma is rare but potentially lethal. Several patients (43.5% in our study) present late with extraocular disease. In high income countries (HICs), children with extraocular disease constitutes less than 5% of cases, whereas it is more than half in low middle income countries (LMICs).^12^ Meanwhile, Gao J et al.^13^ reported a much lower incidence of extraocular condition (8.7%) in South West China.

Non-survivors were more likely to have metastasis and extraocular disease than survivors (66.7% vs 5.4% and 90.6% vs 18.3% respectively). A similar high proportion of advanced disease at presentation was observed in India^14^ and other LMICs.^15^ In HICs^16^ the incidence of extraocular Rb is less than 5% of all cases, attributable to screening protocols for Rb and early referral systems.

The higher frequency of metastasis in extraocular disease reflects the importance of early disease detection to improve survival. Retinoblastoma had metastasized in 26.9% of our cases, greater than in a recent Pakistani study which showed metastasis in 10.8%.^3^ In contrast in Sudan, a much higher percentage of metastasis (44%) in Rb pediatric patients is reported.^17^ The survival rate at TIH is 64.5%, which is within the estimated range of 60 to 92% for other LMIC.^8^ Our study showed a higher prevalence of unilateral retinoblastoma (59.1%) which is consistent with the literature.^3^,^13^,^18^,^19^

In our cohort, chemotherapy was given to 80.6% of patients, EBRT to 29.3% and enucleation was performed in 46.2% patients. Chang et al showed that 47% of cases underwent enucleation while 51% of cases got adjuvant systemic chemotherapy. None of these children showed a tumor recurrence during an average follow-up of 3 years.^20^ Our overall recurrence rate was 68.6% despite management agreed by multidisciplinary decision making. Recurrences were seen after enucleation elsewhere (n=34, 37.6%). This could be because Rb management can be fragmented in Pakistan. The lack of coordinated care due to the dearth of dedicated multidisciplinary panels may cause incorrect staging and subsequently complicate case progression. The presence of a multidisciplinary tumour panel for Rb at TIH includes ophthalmologists, oncologists, radiologists and histopathologists who discuss each case. This may explain why a higher proportion of patients undergoing enucleation at our centre survived (93% vs. 40%).

Meanwhile, the proportion of patients receiving EBRT was higher in non-survivors (48.5% vs. 18.6%). EBRT in our cohort was used when^1^ patients have extraocular Rb or if disease is intraocular, post enucleation histopathology shows high-risk features and^2^ for best supportive care in those with advanced disease to lessen their misery.

In our study, bilateral disease was higher in survivors as compared to non-survivors (41.7% vs. 27.3%). This may be due to heritable Rb, which usually presents with bilateral disease, early in life. Timely recognition and prompt referrals enable a better prognosis.^21^ Patients with leukocoria had better survival, possibly due to an earlier presentation.

Extraocular, recurrent disease and metastasis were found to be significant factors associated with death. Chawla et al.^14^ presented similar results wherein age at presentation, lag time period and the staging of Rb were observed to be associated with worse survival outcome and on further multivariate analysis, staging was found to be associated with survival outcome. Delayed presentation, probably due to a lack of awareness, is cited as a major cause of decreased survival in LMICs.^9^

This study is retrospective and based on a single, albeit tertiary centre. However, the findings in our study help identify important characteristics in the detection and management of patients with retinoblastoma associated with poor outcomes. We recommend screening at birth or at the time of vaccinations where trained paramedics can induce a red reflex and detect any abnormality to be referred to specialists. We plan to initiate awareness campaign at a national level in various forms including social media, electronic media and print media. We propose that any child with signs consistent with Rb be referred to an ophthalmologist to have detailed examination with further multidisciplinary care as appropriate. A multi-level approach at the national, institutional and local community level is important to bring focus to this rare but life-changing and even lethal condition.

## CONCLUSION

There is a high frequency of advanced retinoblastoma including recurrent cases at TIH with a high associated mortality. Whilst all cases were managed with a multidisciplinary approach, some had prior interventions and missed the benefit of this from the outset.

### Authors’ Contribution

**NZ:** Conceived the idea, designed the proposal, writing & editing of manuscript, is responsible for integrity of research.

**AH:** Conceived the idea, designed the proposal

**SI:** Statistical analysis, results write-up, formatting, writing & editing of manuscript

**MHQ &AJ,** Did data collection

**MRK-** Contributed to final concept and design. Drafted, formatted and edited the manuscript.
